# Positively charged residues at the channel mouth boost single-file water flow†
†Electronic supplementary information (ESI) available: Fig. S1: alignment of hAQP1, AQPZ and rAQP4; Fig. S2: SDS-PAGE gel of purified AQP4-eGFP fusion proteins; Fig. S3: determination of reconstitution efficiency; Fig. S4: radius distribution of AQP4 containing PLs; Table S1: charged amino acids located within 15 Å to AQP’s pore entrances and exits. See DOI: 10.1039/c8fd00050f


**DOI:** 10.1039/c8fd00050f

**Published:** 2018-04-02

**Authors:** Andreas Horner, Christine Siligan, Alex Cornean, Peter Pohl

**Affiliations:** a Institute of Biophysics , Johannes Kepler University Linz , Gruberstr. 40 , 4020 Linz , Austria . Email: andreas.horner@jku.at

## Abstract

Water molecules lose two of their four bulk neighbours when entering single-file channels. This process may be sensitive to the presence of positive and negative charges at the channel mouth, since the costs for dehydrating cations and anions differ by a large margin. However, it is not known whether entrance charges affect the single channel water permeability (*p*_f_). So far, *p*_f_ is only known to be governed by H-bond formation between permeating water molecules and wall-lining residues. Here we compare the *p*_f_ values of five different aquaporin species (AQP1, AQPZ, AQP4 wild type, and two phosphorylation mimicking AQP4 mutants) that offer the same number of hydrogen bond donating and receiving residues in their single-file region but display different entrance charges. The *p*_f_ measurements were performed with reconstituted lipid vesicles. We assessed (i) the osmotically induced vesicle deflation from the light scattering intensity in a stopped-flow device and (ii) the aquaporin abundance by fluorescence correlation spectroscopy. Substitution of serine at positions 111 and 180 in AQP4 for aspartic acid showed only a marginal effect on *p*_f_, suggesting that negative entrance charges are of minor importance. In contrast, the total number of positively charged amino acid side chains at entrances and exits correlates with *p*_f_: a total of three, four and seven charges of AQP4, AQPZ, and AQP1 translate into *p*_f_ values of 1.1, 1.8, and 3.2 × 10^–13^ cm^3^ s^–1^, respectively. Thus, positive interfacial charges boost the *p*_f_ value of AQP1 to three times the value of AQP4. Nevertheless, the number of hydrogen bond donating and receiving residues in the single-file region remains the major determinant of *p*_f_. Their effect on *p*_f_ may be a hundredfold larger than that of interfacial charges.

## Abstract 2

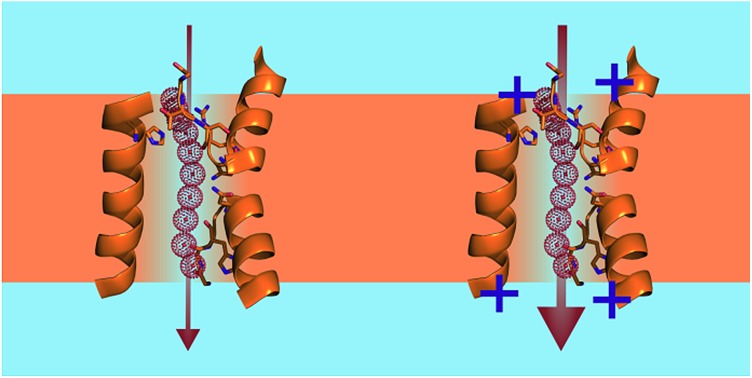
Positively charged residues in the vicinity of the channel entrance or exit accelerate single-file water flow.

## Introduction

Transport through extremely narrow membrane channels plays a crucial role in excitable tissues. The lumen of most excitatory potassium, sodium or calcium channels is too small to let ions and water molecules pass each other. Aquaporins are equally narrow channels, which make major contributions to the maintenance of water homeostasis. Out of proportion to the biological significance of these single-file water channels, our understanding of the underlying physical laws governing single-file transport is very limited.

There is agreement that (i) confinement of water by pore geometry to a one-dimensional file of molecules alters its physico-chemical properties and (ii) movement of water through very narrow membrane channels is different from Poiseuille flow through macroscopic tubes.^1^ Models that assume frictionless flow through the single-file region^2^–^4^ cannot explain why unitary water channel permeability (*p*_f_) varies over three orders of magnitude:^5^ frictionless flow requires the dehydration energy penalty at the channel’s mouth to be the main barrier. However, this barrier is ubiquitous in single-file transport, since water molecules always lose two of the four bulk neighbours upon entering a channel. Only the hydrogen bonds to the preceding water molecule and to the following water molecule remain. This enables the intraluminal water molecules to form new hydrogen bonds with pore-lining residues. The resulting interactions render the flow everything but frictionless. They are responsible for the observed dependence of *p*_f_ on the length of the single-file region.^6^ It is not the number of water molecules in single-file^7^ but the number of hydrogen bond accepting or donating pore lining residues that govern the permeability barrier of the individual channel to water flow.^8^,^30^ Their variability causes *p*_f_ to vary over two or three orders of magnitude.^8^

This observation raises the question of how the two energetic barriers, the dehydration penalty at the entrance and the intraluminal barrier, relate to each other. In other words, does the intraluminal resistance dominate permeation so much that the costs for water dehydration at the channel entrance are negligibly small? To address this question, we exploited the observation that the hydration energies of positive and negative ions are different. Positively charged amino acid side chains (derivatives of ammonium) are weakly hydrated – very much in contrast to their negatively charged counterparts (carboxylates).^9^,^10^ In the case of a non-negligible dehydration penalty, placing positively charged amino acids at the channel entrance may hasten the exchange between water molecules at the pore’s outer hydration shell and the single-file region. In turn, *p*_f_ should increase.

**Fig. 1:**
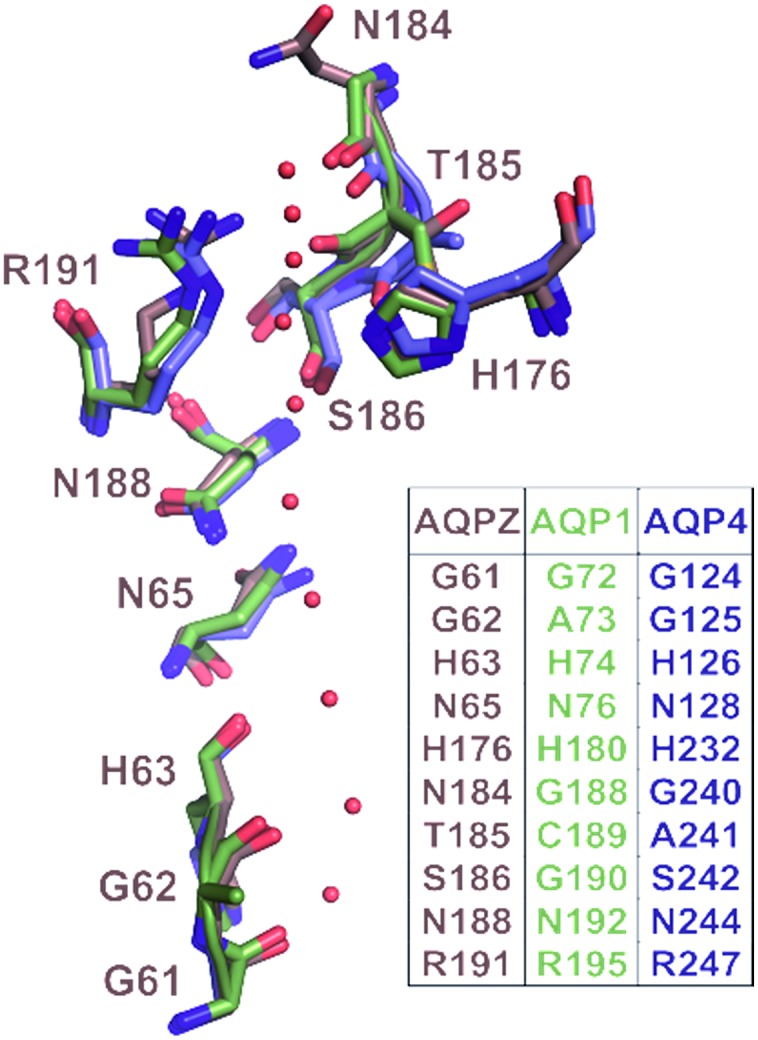
Correlation of pore geometry and potential H-bond forming residues in AQP1, AQPZ and AQP4. The sub-angstrom resolution structure of yeast AQP1 [Protein Data Bank (PDB) #3Z0J] served as a template for the alignment of the AQP1 (PDB #; 1J4N), AQPZ (PDB #; 1RC2) and AQP4 (PDB #; 3GD8) structures *via* the PyMol’s “align” routine. The position of the single-file water molecules (red spheres) in the pore are taken from the yeast AQP1 structure.

**Fig. 2:**
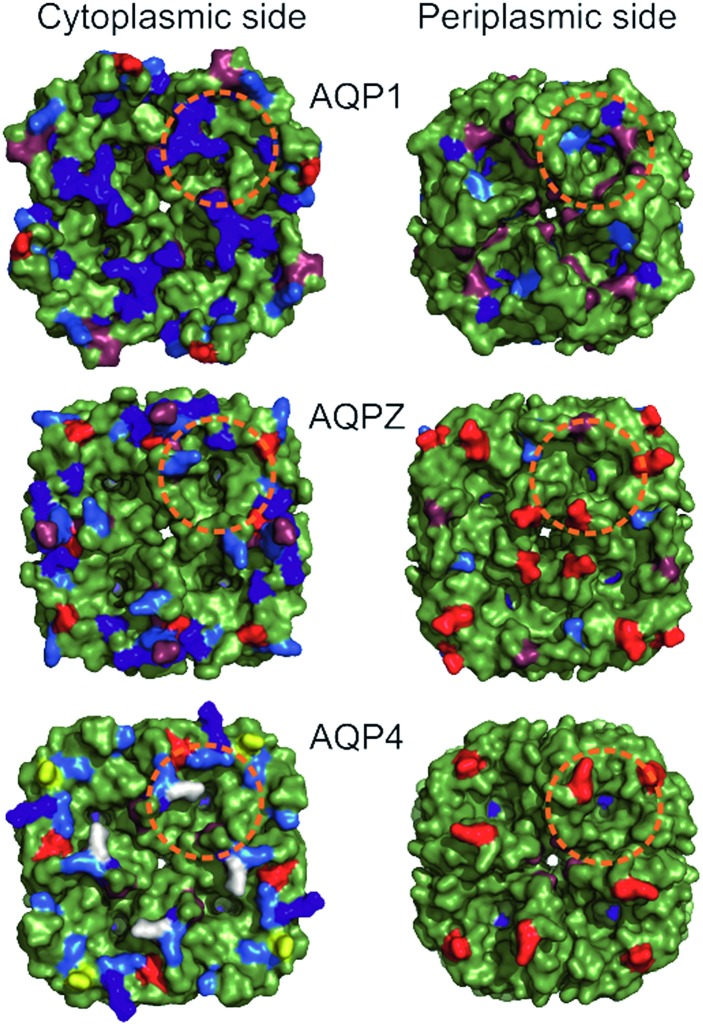
Comparison of periplasmic and cytoplasmic AQP surfaces. Surface representation (green) of AQP1 (PDB #1J4N), AQPZ (PDB #; 1RC2) and AQP4 (PDB #; 3GD8) was achieved using PyMol. Charged amino acid residues located at the periplasmic (Per) and cytoplasmic (Cyt) side are depicted in color (Arg in blue, Lys in marine blue, Glu in red, Asp in raspberry red). The two serines at positions 111 and 180 involved in AQP4 phosphorylation are colored in yellow and white, respectively. Charged amino acid residues (red and blue coloring) are corrected due to a sequence mismatch between the organism of the available high resolution AQP structures used and our purified proteins (AQP1: structure – *Bos taurus*, protein – human; AQP4: structure – human, protein – rat). The orange dashed circles indicate the base of a half-sphere with a radius of 1.5 nm, the regions where charges might influence water molecules entering or exiting the single-file water pore.

We chose five different aquaporin (AQP) species with similar pore regions for this analysis. That is, both the length of the single-file region and the number of potential hydrogen bond forming pore residues are identical for all proteins (Fig. 1). In contrast, the charge densities at the channel entrances differ (Fig. 2): AQP1 has the highest number of positive charges, followed by AQPZ, and AQP4 has the smallest number. AQP4 may be phosphorylated, which would place negative charges at the entrance. It is under debate^13^,^14^ as to whether phosphorylation^11^,^12^ alters *p*_f_. To clarify the potential role of phosphorylation, we measured the *p*_f_ values of wild-type aquaporin-4 and two phosphorylation mimicking mutants in a well-defined reconstituted system that allowed precise control over (i) the aquaporin membrane abundance and (ii) the vesicle volume during deflation.^8^ We compare the *p*_f_ values of the three AQP4 species with those of AQP1 and AQPZ, which we previously reported. While negative charges seem to have no impact on *p*_f_, positive charges at the entrances and exits do augment *p*_f_. This observation indicates that both the dehydration penalty and the intraluminal resistance must be considered when seeking to maximize *p*_f_.

## Results

Taking the 0.88 Å resolution structure of yeast Aqy1 (ref. 15) as a template and using PyMol^16^ to construct homology models of the single-file pore for AQP1, AQPZ, and AQP4 (Fig. 1), we did not find any major structural differences in the pore geometry. However, an analysis of the cytoplasmic and periplasmic vestibules revealed very different charge densities close to the channel mouth (Fig. 2). The charged amino acid residues that locate within a radius of 5 × 3 Å from the first water molecule next to a single-file water molecule are listed in Table S1† and highlighted in Fig. S1.† This approach takes into account that surface induced mobility changes commonly decay within the first five water layers.^17^ Consequently, more distant residues are unlikely to affect dehydration of pore water molecules.

**Fig. 3:**
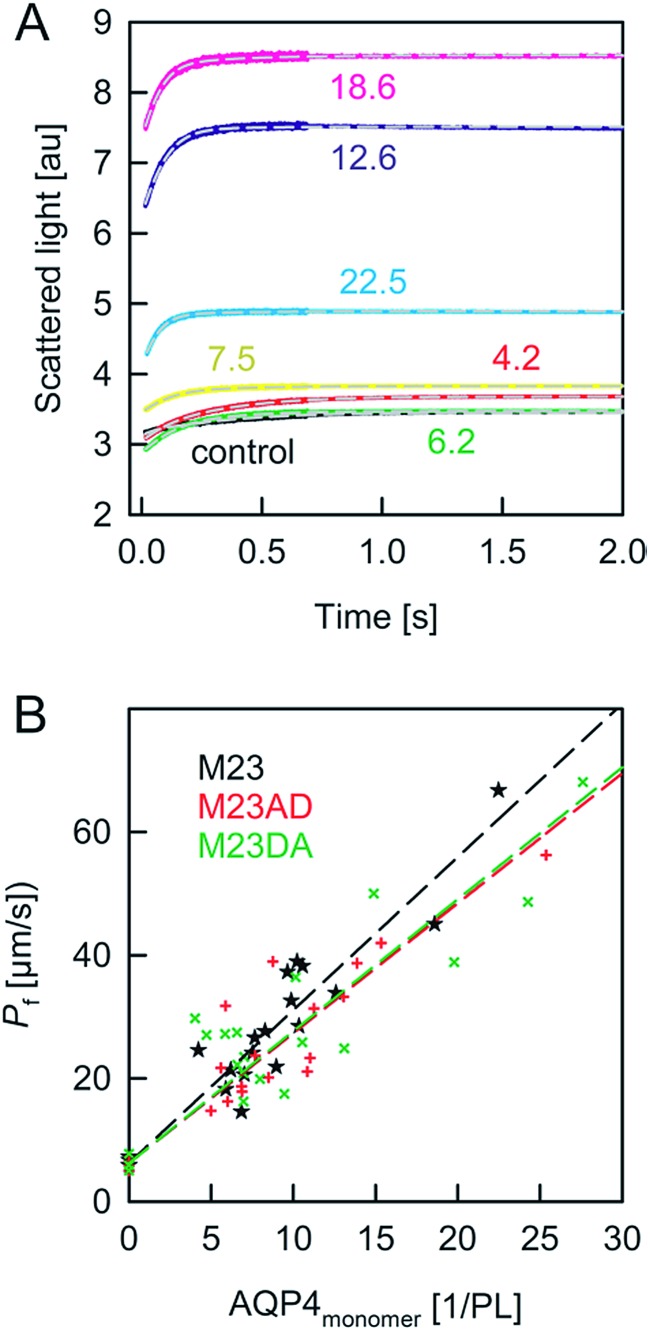
Osmotic shrinkage of AQP4 containing proteoliposomes. (A) Exemplary stopped-flow raw data (colored lines) and fits (short dashed grey lines) according to eqn (1) and (2). A vesicle suspension and hyperosmotic solution (300 mM sucrose) were mixed at equal volumes (4 °C, 100 mM NaCl, 20 mM MOPS, pH 7.5). The number of AQP4M23 monomers per PL is indicated. (B) The *P*_f_ value of the proteoliposomes was calculated according to eqn (1) and (2) from the data shown in (A) and plotted as a function of the number of AQP monomers per proteoliposome. The data for AQP4M23 and phosphorylation mutants consist of three independent purifications and reconstitutions each.

**Fig. 4:**
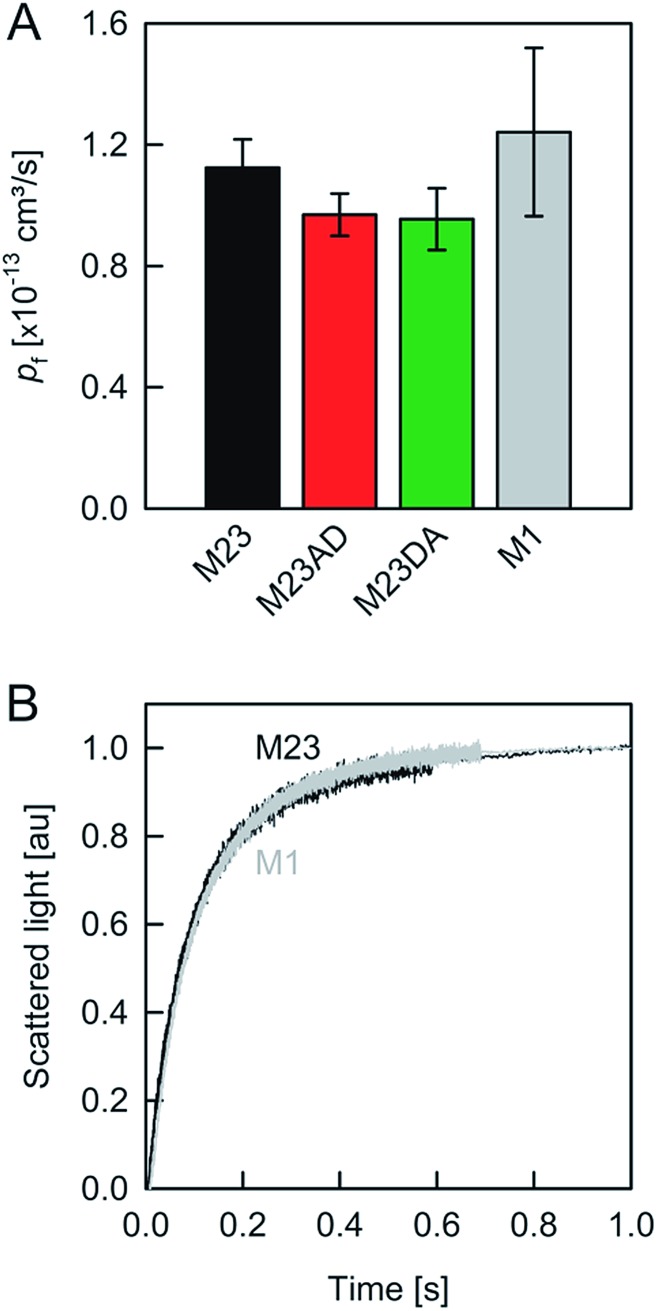
Water movement through APQ4. (A) *p*_f_ (at 4 °C) was calculated from the slopes of linear regressions to the data in Fig. 2B. (B) M1, the longest isoform of AQP4, and M23 exhibit similar *p*_f_ values.

We started our experiments with overexpression, purification and reconstitution of AQP4M23, an isoform which is thought to assemble into orthogonal arrays of particles (OAPs).^18^,^19^ After reconstitution into lipid vesicles, we exposed the proteoliposomes (PLs) to a hyperosmotic solution. We monitored PL deflation by recording the scattered light intensity (Fig. 3A), which served to calculate the vesicle volume *V*(*t*) *via* an adaptation of the Rayleigh–Gans–Debye equation.^8^ In turn, *V*(*t*) allowed computing the vesicle permeability *P*_f_ (eqn (2)). Increasing the membrane abundance of AQP4M23 per PL accelerated the rate of vesicle shrinkage (Fig. 3A). Plotting *P*_f_ as a function of *n* (Fig. 3B) served to derive *p*_f_ from the slope of a linear fit. With only 1.1 ± 0.1 × 10^–13^ cm^3^ s^–1^ at 4 °C (Fig. 4A), *p*_f_ of reconstituted AQP4M23 amounts to only 1/3 of AQP1’s *p*_f_ value.

To preclude OAP formation from hampering the comparison to other aquaporins, we also performed experiments with M1, the longer AQP4 isoform, which lacks the OAP forming ability of AQP4M23. In our system, AQP4M1 matched the *p*_f_ value of AQP4M23 (Fig. 4). This observation is in line with our assumption that the limited number of available AQP4 molecules per vesicle renders OAP formation unlikely.

**Fig. 5:**
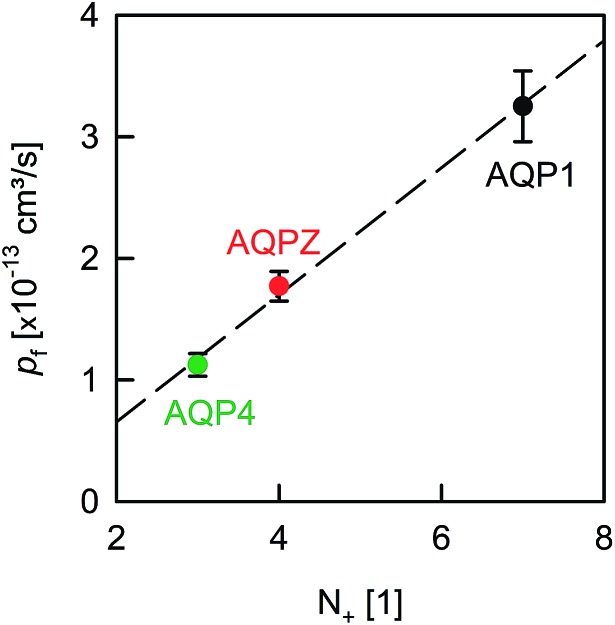
Dehydration penalty at the channel mouth. *p*_f_ depends on the amount of positively charged amino acid residues (*N*_+_) near the pore entrances. The *p*_f_ values of AQP1 and AQPZ are taken from our previous report.^8^*N*_+_ is estimated from Fig. 2 (also compare in Table 1).

A comparison of the *p*_f_ value of AQP4 with our previously reported AQP1 and AQPZ values, which we obtained in a similar fashion, reveals a highly significant correlation between *p*_f_ and the number of positive charges at the channel mouth (*N*_+_) (Fig. 5). Neither the net charge nor the number of negative charges seem to correlate with *p*_f_ (Table 1). To validate this hypothesis, we performed measurements with two phosphorylation mimicking mutants that introduce one additional negative charge to the cytoplasmic entry. Aspartates or alanines replaced the phosphorylation target sites at positions 111 and 180 (S111A S180D = AD mutant; S111D S180A = DA mutant). Similar experiments as described above revealed only a minor decrease in *p*_f_ of less than 15% for both mutants (Fig. 4A), indicating that only positively charged protein residues affect *p*_f_.

**Table 1:** Charge distribution at the AQP’s pore entrances. Comparison of the amount of arginines, lysines, aspartic acids and glutamic acids located within 5 water layers from the first water molecule next to the single-file water molecules at the periplasmic (Per) and cytoplasmic (Cyt) side of hAQP1, AQPZ, rAQP4 M1 and M23. The star indicates our phosphorylation mutants where we substituted Ser111 and Ser180 for Ala and Asp. The total number of positively or negatively charged amino acid residues is depicted as *N*_+_ and *N*_–_, respectively. *N*_±_ indicates the net charge

	AQP1		AQPZ		AQP4M23		AQP4M23*		AQP4M1	
	Per	Cyt	Per	Cyt	Per	Cyt	Per	Cyt	Per	Cyt
Arg	1	4	—	2	—	1	—	1	—	1
Lys	1	1	—	2	—	2	—	2	—	2
Asp	3	3	—	1	1	2	1	3	1	2
Glu	—	—	1	1	1	1	1	1	1	1
*N*_+_	2	5	—	4	—	3	—	3	—	3
	7		4		3		3		3	
*N*_–_	3	3	1	2	2	3	2	4	2	3
	6		3		5		6		5	
*N*_±_	1	2	1	2	2	—	2	1	2	—
	1		1		2		3		2	

The correlation between *N*_+_ and *p*_f_ shows a lower entrance barrier in the case of weakly hydrated entrance residues. This observation may help to optimize the performance of artificial water channels.

## Discussion

We have found clear evidence for the contribution of the dehydration energy to the total energy barrier of water permeation across channels. That is, the *p*_f_ value of AQPs increased threefold upon increasing the amount of positively charged amino acid residues located at the channel entrances (Fig. 5). Thus, the effect of changing the barrier at the entrance is comparable in size with the deletion of a few residues that donate or receive hydrogen bonds in the channel wall. We conclude that both the charges at the entrance and the hydrogen bonds within the channel make significant contributions to the single-file water mobility.

Dissecting the roles of dehydration and rehydration would require a non-random orientation of the reconstituted aquaporins. If dehydration was limiting, a sixfold *p*_f_ increase due to surface charges could be derived from the observed threefold increase for the average water permeation rate in both directions. We conclude that modulation of the entrance barrier answers for a comparatively small variability in single-file *p*_f_, as the effect does not exceed one order of magnitude. In contrast, adding or deleting hydrogen bond donating and receiving residues to/from the pore lining has been shown to exert a roughly hundredfold larger effect.^8^

The measured correlation between *p*_f_ and *N*_+_ is in line with the observation that positively charged residues are weakly hydrated – very much in contrast to their negatively charged counterparts.^20^ However, it does not agree with observations made for surfaces adjacent to extended aqueous solutions: close to a hydrophilic surface, transient binding or trapping of water molecules over times of hundreds of picoseconds was reported. Only in the vicinity of hydrophobic surfaces was water dynamics purely diffusive.^21^ The reason for this discrepancy is not entirely clear. It is certain that the extreme confinement plays a role, as has been shown for the example of protein crowding which induced collective hydration of biological macromolecules over extended distances.^22^ It has also been shown that hydrogen bonds have a bigger lifetime in general – but the effect that individual amino acid residues may have on the collective H-bond lifetime is not known.

Our results also have implications for brain physiology since AQP4 is believed to be the major player in brain water homeostasis. The water channel protein was long believed to be gated – phosphorylation of Ser180 by protein kinase C being the trigger for channel closure.^11^ Conversely, phosphorylation of Ser111 in loop B was reported to activate AQP4 (ref. 12, 23 and 24). We now have shown that the corresponding phosphorylation mimicking mutants did not display a change in *p*_f_ – *i.e.* neither closure nor activation occurred. Our result is in line with the observations (i) of rat astrocyte primary cultures where AQP4 activity was independent of phosphorylation;^25^ (ii) that mutation of Ser111 to alanine or aspartate does not change *p*_f_;^14^ (iii) where molecular dynamics simulations showed only a marginal effect of Ser180 phosphorylation on *p*_f_;^13^ and (iv) of a lack of conformational differences between the wild type and the S180D mutant.^18^,^26^

We conclude that positively charged residues in the immediate vicinity of the mouths to single-file pores serve to boost *p*_f_. Conceivably, the effect is mediated by a reduction in the collective H-bond lifetime in the single-file region. Future experiments, in which the orientation of AQP reconstitution is controlled, may serve to prove that hypothesis.

## Experimental

### Materials and methods

#### Cloning and expression

Coding sequences of rat AQP4M23 were cloned upstream a C-terminal EGFP into pYES2 yeast expression vectors. Using site-directed mutagenesis, putative serine phosphorylation sites of rAQP4, S111 and S180 were mutated to obtain phosphorylation mimicking rAQP4 S111A S180D and rAQP4 S111D S180A forms of AQP4. All aquaporins were transformed into *S. cerevisiae* InvSc (invitrogen) and grown to an OD of 1.5 in DOB-Ura selective medium at 30 °C before inducing the overexpression of the proteins in YPG medium overnight at 30 °C. Cells were harvested and pellets frozen at –80 °C.

#### Protein purification and reconstitution

Purification of the aquaporins was performed as previously described^27^ with minor modifications (Fig. S2†). In short, cell pellets were lysed in 100 mM K_2_HPO_4_ at pH 8.0 in the presence of protease inhibitors (complete-EDTA) and subjected to 3 cycles of French press (20 000 psi). The lysate was recovered after 10 min of 4516 × *g* and membrane fractions were pelleted by centrifugation at 99 187 × *g* at 4 °C for 80 min. The membrane fractions were solubilised in buffer A (100 mM K_2_HPO_4_, 200 mM NaCl, 10% glycerol, 5 mM β-mercaptoethanol, proteinase inhibitor complete-EDTA) containing 20 mM imidazole at pH 8.0. After 1 h of incubation with 3% OG, insoluble material was pelleted by centrifugation at 40 000 rpm at 4 °C for 1 h. The supernatants were incubated with 2 ml of equilibrated NiNTA-beads overnight at 4 °C. The beads were packed onto columns and washed with 200 ml of buffer A in the presence of 100 mM imidazole at pH 7.0. The NiNTA-bound proteins were eluted 5 times with 0.5 ml of buffer A containing 1 M imidazole at pH 7.0.

AQP4 was reconstituted into proteoliposomes (PLs) as previously described.^8^ In brief, *E. coli* polar lipids (PLE, Avanti Polar Lipids) doped with 0.004 m% Atto633PPE were dried on a rotary evaporator, rehydrated in reconstitution buffer (100 mM NaCl, 20 mM MOPS, 1.4% OG, pH 7.4) and bath sonicated. The clear 20 mg ml^–1^ suspension was incubated with equal amounts of protein diluted in reconstitution buffer at room temperature for an hour. After step-wise removal of the detergent with Biobeads SM-2 (Bio-Rad) over 36 hours at 4 °C, the PLs were harvested by ultracentrifugation. The resuspended vesicles were put through 21 extrusion cycles stacked with two polycarbonate filters with 100 nm pore sizes using a mini-extruder from Avanti Polar Lipids and centrifuged to remove aggregates. Control vesicles were treated similarly. All samples were assayed without delay.

#### Determination of unitary water permeabilities (*p*_f_)

The PLs and LUVs were subjected to a hyperosmotic solution in stopped-flow apparatus (SFM-300, Bio-Logic, Claix, France) at 4 °C. As previously described, we monitored the intensity of the scattered light at 90° at a wavelength of 546 nm.^28^ The integral water permeability (*P*_f_) of the LUVs was obtained by fitting eqn (1) to the experimental data:^8^,^31^

1 *I*(*t*) = *a* + *b*[*αV*_bare_(*t*) + (1 – *α*)*V*_AQP_(*t*)] + *d*[*αV*_bare_(*t*) + (1 – *α*)*V*_AQP_(*t*)]^2^

where *α* in eqn (1) is the fraction of bare vesicles, which did not contain AQP4. *V*(*t*) depends on *P*_f_ according to:^8^,^31^
2
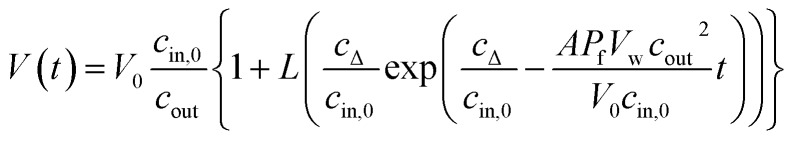
where *V*_w_, *V*_0_, *A*, *c*_in,0_, *c*_out_, *c*_Δ_, and *L* are the molar volume of water, vesicle volume at time zero, surface area of the vesicle, the initial osmolyte concentration inside the vesicles, the outer and the incremental osmolyte concentration in the external solution due to sucrose addition, and the Lambert function *L*(*x*)e^*L*(*x*)^ = *x*, respectively. We globally fitted eqn (1) and (2) to all data from one reconstitution batch. The fit took into account the variability in *V*_0_ (Fig. S4†).

#### Determination of channel density per proteoliposome

Calculation of *p*_f_ requires the surface density of the AQP4 molecules to be known. Therefore, we exploited fluorescence correlation spectroscopy (FCS) to determine the number *n* of AQP monomers per proteoliposome, as previously described.^29^,^32^ In brief, recording the fluctuations in the fluorescence intensity *I* in the protein channel allowed calculation of the autocorrelation curve (Fig. S3†):3
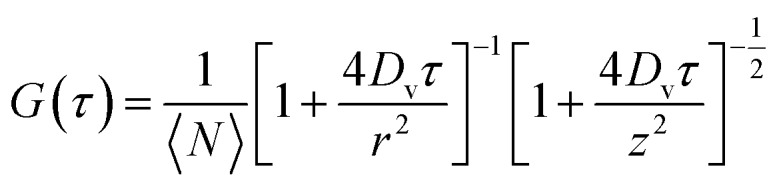
where where 〈*N*〉, , *z*, *r*, *D*_v_ are the number of particles in the focal volume, the elongation of the focus in the direction of the laser beam and perpendicular to it, and the diffusion coefficient of the fluorescent particles, respectively. First we obtained the number *n*_PL_ of proteoliposomes per focal volume *via*eqn (3). Subsequently, we dissolved the PLs in detergent (2% OG + 2% SDS) and calculated the number of protein containing micelles (*n*_m_) per unit volume and their brightness (*I*_micelle_). Hence, *n* can be calculated by the ratio (*n*_m_*F*)/*n*_PL_, with *F* being the correction factor for undissolved oligomers in the detergent solution. *F* was determined by the ratio *I*_micelle_/*I*_monomer_. The brightness of the AQP containing micelles and the single AQP4 monomers was measured under similar buffer conditions.

## Conflicts of interest

There are no conflicts to declare.

## Supplementary Material

Supplementary information
