# Supplementary material for: Positively charged residues at the channel mouth boost single-file water flow
Source: Faraday Discuss. 2018 Apr 2;209(0):55–65. doi: 10.1039/c8fd00050f (PMC6161257; doi:10.1039/c8fd00050f)
Supplement: Supplementary file 1 [file FD-209-C8FD00050F-s001.pdf]

Electronic Supplementary Material (ESI) for Faraday Discussions.
This journal is © The Royal Society of Chemistry 2018

Supplementary Information

Positively charged residues at the channel mouth boost single-file water flow

Andreas Horner[a,*,#], Christine Siligan [a,*], Alex Cornean [a,b], Peter Pohl[a]

[a] Institute of Biophysics, Johannes Kepler University Linz, Gruberstr. 40, 4020 Linz, Austria.

[b] Current address: Centre for Organismal Studies (COS), Heidelberg University, Im Neuenheimer Feld 230, 69120 Heidelberg, Germany.

[#] Correspondence should be sent to andreas.horner@jku.at

* Authors contributed equally to the work.

```
                 N_Cyt                              H1
AqpZ    --------------------------------MDMFRKLAAECFGTFWLVFGGCSAVLAA
hAqp1   ------------------------MASEFKKKLFWRAVVAEFLATTLFVFISIGSALGFK
rAqp4   MSDGAAARRWGKCGPPCSRESIMVAFKGVWTQAFWKAVTAEFLAMLIFVLLSVGSTINWG
                                          ::: :.** :.    :*: . **::

             L_per1          H2           L_Cyt2     HB       L_Cyt3
AqpZ    G----FPELGIGFAGVALAFGLTVLTMAFAVGHISGGHFNPAVTIGLWAGGRFPAKEVVG
hAqp1   YPVGNNQTAVQDNVKVSLAFGLSIATLAQSVGHISGAHLNPAVTLGLLLSCQISIFRALM
rAqp4   G---SENPLPVDMVLISLCFGLSIATMVQCFGHISGGHINPAVTVAMVCTRKISIAKSVF
                   . . ::*.***:: *:. ..*****.*:*****:.:   ::   . :

             H3                         L_Per4               H4
AqpZ    YVIAQVVGGIVAAALLYLIASGKTGFDAAASGFASNGYGEHSPGGYSMLSALVVELVLSA
hAqp1   YIIAQCVGAIVATAILSGITSSLTGNSLGRNDLA---------DGVNSGQGLGIEIIGTL
rAqp4   YITAQCLGAIIGAGILYLVTPPSVVGGLGVTTVH---------GNLTAGHGLLVELIITF
        *: ** :*.*:.:.:*  ::   .  . . . .           .. .   .* :*:: :

          H4       L_Cyt5         H5            L_Per6    HE      L_Per7
AqpZ    GFLLVIHGA--TDKFAPAGFAPIAIGLALTLIHLISIPVTNTSVNPARSTAVAIFQGGWA
hAqp1   QLVLCVLATTDRRRRDLGGSAPLAIGLSVALGHLLAIDYTGCGINPARSFGSAVITHNFS
rAqp4   QLVFTIFASCDSKRTDVTGSVALAIGFSVAIGHLFAINYTGASMNPARSFGPAVIMGNWE
         ::: : .:     :     * . :***:::: **::*  *. .:*****  . *::  .:

             H6                                 C_Cyt
AqpZ    LEQLWFFWVVPIVGGIIGGLIYRTLLEKRD------------------------------
hAqp1   --NHWIFWVGPFIGGALAVLIYDFILAPRSSDLTDRVKVWTSGQ--VEEYDLDADDINSR
rAqp4   --NHWIYWVGPIIGAVLAGALYEYVFCPDVELKRRLKEAFSKAAQQTKGSYMEVEDNRSQ
          : *::** *::*. :.  :*  ::

AqpZ    -------------------------------------
hAqp1   VE-----MKPK--------------------------
rAqp4   VETEDLILKPGVVHVIDIDRGDEKKGKDSSGEVLSSV
```

Figure S1. Alignment of hAQP1, AQPZ and rAQP4. The N- and C-termini are located both at the cytoplasmic side of the protein. Transmembrane helices H1 to H6, half-helices HB and HE as well as the location/orientation of loop L1 to L7 are indicated. Charged amino acid residues are labeled according to the color rules defined in Fig. 2. C- and N-terminal amino acids resolved in the respective structures are labeled in yellow. Potential H-bond forming residues located in the single-file pore region in AQP1, AQPZ and AQP4 are highlighted in green. M1, M23, Ser111 and Ser180 as

well as all charged amino acid residues which are located in the region of interest (Fig. 2) are depicted in bold letters. The multiple sequence alignment was performed with Clustal O(1.2.4).

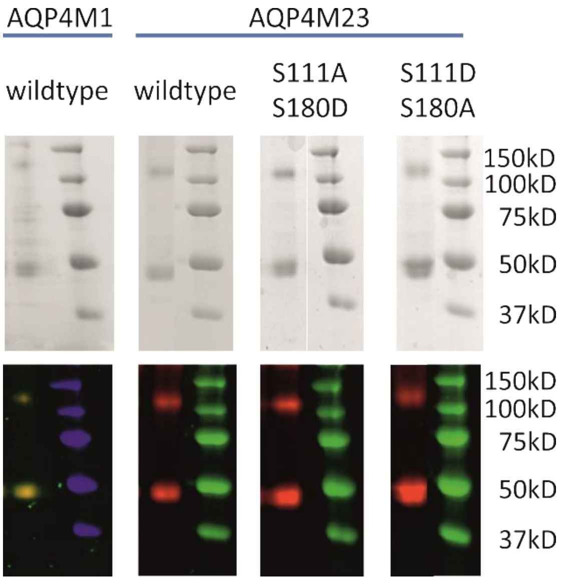

Figure S2. SDS-PAGE gel of purified AQP4-eGFP fusion proteins. Upper panel: Coomassie-stained gel showing purified mono- and oligomeric AQP4-eGFP at ~50kDa and ~120kDa, respectively. Lower panel: fluorescent imaging of AQP4-eGFP proteins.

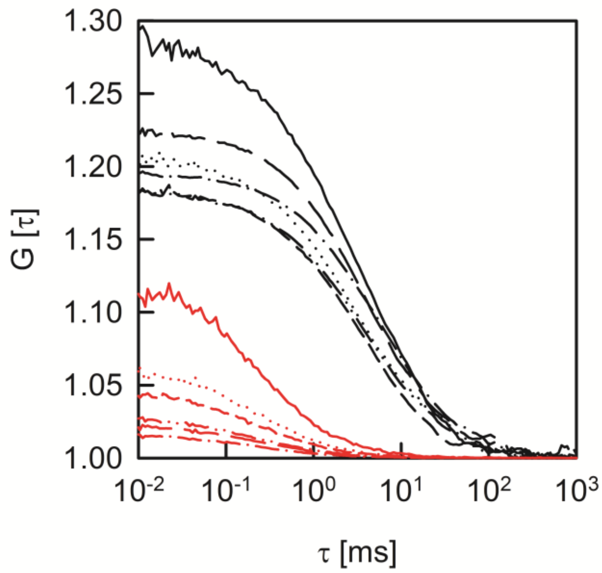

Figure S3. Determination of reconstitution efficiency. Representative FCS autocorrelation curves of an AQP4M23 reconstitution series as depicted in Fig. 3, A. PL (black lines) are dissolved in detergent (2% OG + 2% SDS) to form AQP4M23eGFP containing micelles (red lines). The ratio between AQP4 containing micelle and the number of PL serves to calculate the number of AQP4 units per PL.

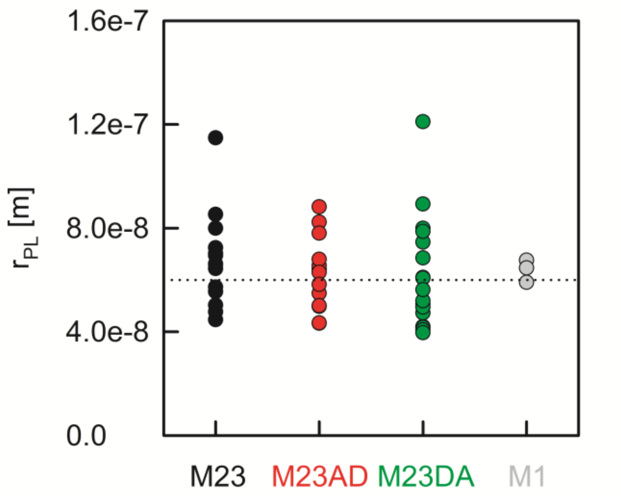

Figure S4. Radius distribution of AQP4 containing PL's. The black dotted line represents PL with a radius r_{PL} of 60nm.

hAQP1		AQPZ		rAQP4	
**K6**	**14.27**	**K6**	**9.26**	D100	11.30
**K36**	**7.90**	E33	14.24	**K140**	**9.08**
**R93**	**8.48**	**R77**	**8.64**	D142	13.26
**R126**	**11.59**	**K81**	**14.22**	**K145**	**7.42**
D128	8.90	E82	10.10	D210	12.82
D131	14.33	D156	12.96	D211	10.45
D158	12.76	**R232**	**14.30**	**R182**	**10.79**
**R159**	**9.14**			D215	9.06
**R161**	**12.25**			E259	13.47
D163	10.26			E280	11.65
D185	8.81				
D228	13.69				
**R234**	**12.79**				

Table S1. Charged amino acids located within 15 Å to AQP's pore entrances and exits. Distances in angstrom are measured in PyMol from the first water molecule next to a single-file water molecule at the periplasmic and cytoplasmic side of hAQP1, AQPZ, rAQP4 to charged amino acid residues. Positively charged amino acids are labeled in blue and negatively charged amino acids in red.
